# Bone erosions and joint damage caused by chikungunya virus: a systematic review

**DOI:** 10.1590/0037-8682-0433-2023

**Published:** 2024-04-05

**Authors:** José Kennedy Amaral, Peter Charles Taylor, Robert Taylor Schoen

**Affiliations:** 1 Instituto de Medicina Diagnóstica do Cariri, Departamento de Reumatologia, Juazeiro do Norte, CE, Brasil.; 2 University of Oxford, Nuffield Department of Orthopaedics, Rheumatology and Musculoskeletal Sciences, Oxford, United Kingdom.; 3 Yale University, School of Medicine, Section of Rheumatology, New Haven, Connecticut, United States of America.

**Keywords:** Chikungunya virus, Rheumatoid arthritis, Disease-modifying antirheumatic drugs, Bone erosion, Joint damage

## Abstract

**Background:**

Chikungunya fever is an emerging global infection transmitted by *Aedes* mosquitoes that manifests as an acute febrile illness with joint pain and can lead to chronic arthritis. The mechanism underlying chronic joint damage remains unclear; however, chronic chikungunya arthritis shares similarities with rheumatoid arthritis. Disease-modifying antirheumatic drugs have revolutionized rheumatoid arthritis treatment by preventing joint damage. However, the role of these therapies in chronic chikungunya arthritis has not been determined. We conducted a systematic review to evaluate the burden of joint structural damage in chronic chikungunya arthritis to help to define the role of disease-modifying therapy in this disease.

**Methods:**

This systematic review included retrospective and prospective studies, trials, and case reports evaluating joint damage caused by chikungunya virus. Various databases were searched without any date or language restrictions. Study selection was conducted independently by two researchers, and data were extracted from the articles selected.

**Results:**

A total of 108 studies were initially evaluated, with 8 meeting the inclusion criteria. Longitudinal studies have reported persistent joint pain from chikungunya infection and the progression of radiographic joint damage up to 13 years post-infection. Joint imaging revealed synovial inflammation, bone erosion, and cartilage destruction in patients with chronic chikungunya arthritis.

**Conclusions:**

Few studies have addressed chikungunya-induced joint damage, limiting our understanding of chronic chikungunya arthritis. Nevertheless, chronic chikungunya arthritis has similarities to rheumatoid arthritis. The success of early disease-modifying antirheumatic drug therapy in rheumatoid arthritis underscores the need for comprehensive research on its role in chikungunya arthritis.

## INTRODUCTION

Chikungunya fever (CHIKF) is an emerging global infection caused by chikungunya virus (CHIKV), an RNA alphavirus transmitted by *Aedes* mosquitoes^1^. Following vector transmission, CHIKF usually presents within a 3-5-day incubation period as an acute febrile illness with severe polyarthralgia/polyarthritis, rash, headache, fatigue, and sometimes nausea, vomiting, and diarrhea. Less commonly, CHIKV infection affects other organs such as the brain, heart, lungs, kidneys, and skin^2^. In recent years, large outbreaks of CHIKF have occurred in the Americas, Asia, Africa, and parts of Europe. In Brazil alone, there have been more than 1 million cases^3^. CHIKF is rarely fatal, but often causes a chronic rheumatic phase known as chronic chikungunya arthritis (CCA), which can last for weeks, months, or years, affecting up to 40% of those infected^4^.

However, the pathogenesis of CCA remains unclear. Actively replicating CHIKV has not been found in CCA synovial tissue and CHIKV surface antigens have not been detected in muscle fibroblasts harboring persistent viral RNA^5^. It has been suggested that CHIKV infection of mesenchymal stem cells (MSCs) may cause epigenetic changes in both MSCs themselves and in their progenies, without the need for reactivation of dormant viruses^6^.

Rheumatoid arthritis (RA) is a chronic systemic autoimmune disease of unknown etiology that affects multiple joints and results in cartilage destruction and bone erosion. Similar to CCA, RA causes a chronic inflammatory process that can damage joints and other organs, including the kidneys, heart, and lungs^7^. The global prevalence of RA is approximately 1%, with a female predominance. The incidence varies by age and population^8^.

**FIGURE 1:**
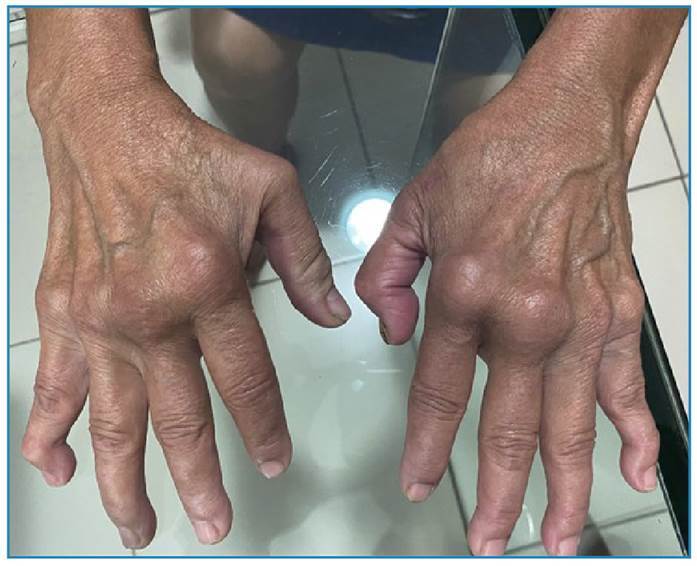
Patient with joint deformities of the hands 6 years after confirmed chikungunya virus infection.

The clinical presentation of CCA can mimic RA (Figure 1). There have been multiple reports of patients with CCA who met the 2010 American College of Rheumatology (ACR) diagnostic criteria for RA^9^. The pathogenesis of CCA and RA may involve similar mechanisms. Both CCA and RA are associated with high circulating levels of pro-inflammatory cytokines, including interleukin 6 (IL-6), granulocyte-macrophage colony-stimulating factor (GM-CSF), interferon alpha (IFN-α), IL-17, and IL-6, are important in joint inflammation and contribute to the production of cartilage-destroying enzymes^9^.

Three decades ago, nonsteroidal anti-inflammatory drugs (NSAIDs) were considered the preferred initial treatment for RA. If these treatments failed, disease-modifying antirheumatic drug (DMARD) therapy was then initiated. However, in the early 2000s, several randomized controlled trials demonstrated that early and aggressive disease-modifying therapy using the most efficacious interventions available is essential to optimally control disease activity and prevent irreversible disease progression^7^ ^,^ ^10^.

It is currently recommended that CCA should initially be treated with analgesics, NSAIDs, and/or corticosteroids. DMARD therapy is recommended only if standard treatments have been used for at least 3 months and have failed^11^ ^,^ ^12^. However, CCA can follow a chronic inflammatory course similar to that of RA with irreversible joint damage RA^13^.

We and others have demonstrated that low-dose methotrexate (MTX) is often effective for CCA^14^. Patients with CCA may benefit from early treatment with MTX or other DMARDs. Just as there has been a paradigm shift in the treatment of RA in recent decades, our observations in CCA suggest that the early use of DMARDs should be an essential aspect of management with the goal of preventing structural damage; however, there is currently no robust evidence on the role of DMARDs in the treatment of CCA^11^ ^,^ ^12^. To further validate this hypothesis, we performed a systematic review of published studies on joint damage caused by CHIKV infection.

## METHODS

### ● Protocol and registration

The study protocol was registered in the International Prospective Register of Systematic Reviews (PROSPERO) (protocol #CRD42023421183.) This study was conducted according to the Preferred Reporting Items for Systematic Reviews and Meta-Analyses (PRISMA)^15^ guidelines to address the following question: "Does chikungunya cause bony erosions and joint damage?"

### ● Eligibility criteria

Retrospective and prospective studies, randomized controlled trials, case reports, and case series reporting joint damage caused by CHIKV infection were included in this review. There were no restrictions based on age, sex, or race. The studies were required to provide clinical and epidemiological confirmation of CHIKV infection. Eligible studies evaluated the presence of joint damage confirmed by imaging tests such as radiography, ultrasonography, computed tomography, or magnetic resonance imaging (MRI).

### ● Information sources and searches

Electronic databases, including MEDLINE (PubMed), Scopus, EMBASE, and the Cochrane Library, were searched through June 5, 2023, without language and date restrictions. In addition, we included studies identified through manual searches conducted using Google Scholar, gray literature, conference summaries, and reference lists. Details of the databases, search dates, search strategies used, and number of retrieved studies can be found in the supporting information (S1 Table).

### ● Study selection

JKA and RTS independently screened titles, abstracts, and full texts to confirm eligibility, and disagreements were resolved by consensus. When this was not possible, a third investigator (PCT) was consulted.

### ● Data collection process and data items

JKA and RTS independently extracted data from the included studies using a standard data extraction form (S2 Table). The form recorded the country and year of study publication, study design, number of participants, diagnosis, and description of bone erosions and joint damage.

## RESULTS

### ● Study selection

A total of 188 studies were retrieved from the database searches. Most studies did not meet our inclusion criteria, and 98 articles were excluded as duplicates. Eight studies met the inclusion criteria and were included in the systematic review.

### ● Study characteristics

**FIGURE 2:**
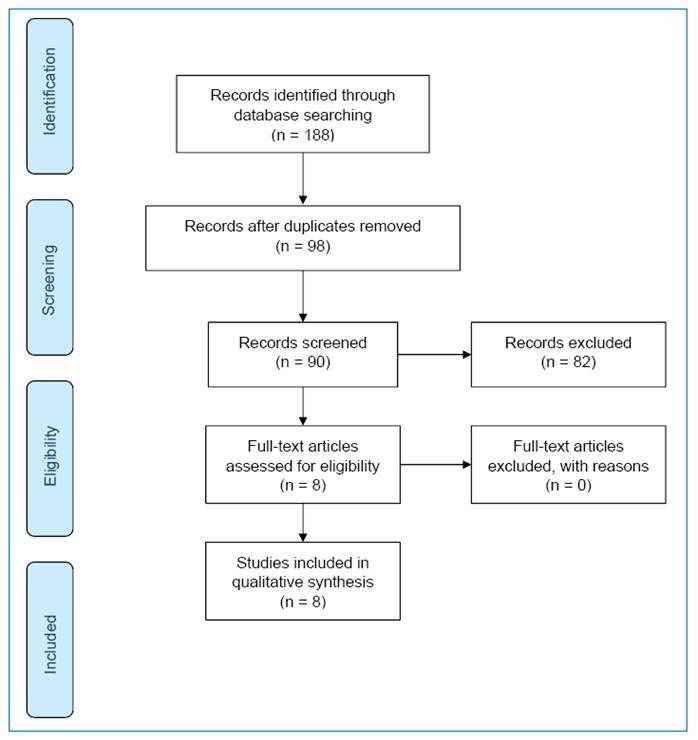
Flow chart of the selection of studies on bone erosions and joint damage caused by chikungunya virus for inclusion in the systematic literature review.

Of the eight studies meeting the eligibility criteria, four were retrospective studies, one was a prospective study, and three were case reports (Figure 2). Three studies were conducted in France or French territories, three in Brazil, one in India, and one in Japan.

### ● Study findings

In a longitudinal observational study, Guillot et al.^16^ used radiography to assess the joints of 30 patients with confirmed chikungunya who were evaluated 13 years after the initial infection. This study included 20 women (mean age 60.1 [57.2-63.1] years). The authors found that patients with persistent joint symptoms had higher CHIKV immunoglobulin M (IgM) and immunoglobulin G (IgG) antibody levels. Three patients with persistent arthritic symptoms remained CHIKV IgM-positive even 13 years after the infection, compared with one asymptomatic patient. Compared with asymptomatic patients, symptomatic patients also had a higher prevalence of radiographic joint damage (29% versus 15%). At 13 years after infection, radiographic damage indicative of inflammatory joint diseases was observed in 7 of 30 patients with CCA compared with 5 of 19 in the RA control group, which might have been reduced by using immunomodulatory drugs^16^.

In a cohort of 94 patients with CHIKV infection, Manimunda et al.^13^ evaluated 20 individuals with persistent joint pain 10 months after acute infection, testing for rheumatoid factor (RF) and anti-cyclic citrullinated peptide (anti-CCP) antibody, and joint imaging with radiography and MRI. None of the 20 patients tested positive for RF, but one tested positive for anti-CCP antibody. Five patients had radiolucent lesions suggestive of bony erosion on radiography. The MRI findings included joint effusion (16/20), bony erosion (4/20), marrow edema/erosion (7/20), synovial thickening (4/20), tenosynovitis (2/20), and tendinitis (3/20). Degenerative joint disease, meniscal tearing, and cruciate ligament damage were also observed.

The case reports and case series found nonspecific but important joint alterations. One case report by Eyer-Silva et al.^17^ described a 36-year-old man infected with CHIKV who developed severe acute arthritis in a finger joint previously damaged by trauma, confirmed on radiography.

Malvy et al.^18^ described the radiographic and MRI changes in a 60-year-old man with CHIKF who developed progressive erosive arthritis. Subchondral defects were observed on radiographic images in the 2nd and 3rd right proximal interphalangeal finger joints and in the 3rd, 4th, and 5th left distal interphalangeal joints. MRI resonance imaging revealed bilateral periosteal inflammation, edematous carpitis, carpal synovitis, bone destruction, and intraarticular swelling. This individual had elevated levels of inflammatory mediators and persistent specific anti-CHIK IgM antibodies over the 24 months following infection.

Mizuno et al.^19^ assessed the subsequent development of arthritis in six Japanese patients (four women aged 36 to 56 years, and two men aged 30 and 52 years) with CHIKV infection. One patient developed MRI-confirmed bone erosions, and another developed extensive carpal styloid erosions .

Bouquillard et al.^20^ reported 21 cases (13 women; mean age, 57 ± 12 years) of RA diagnosed at a rheumatological center on Reunion Island after a CHIKF epidemic in 2005. During a mean follow-up period of 27.6 months, radiography of the hands and feet revealed erosions and/or joint-space narrowing in 12 patients. All patients were treated with DMARDs. Progression of structural damage was observed, with erosion and/or joint space narrowing identified in 17 patients. Nine patients had normal baseline radiographs, but MRI revealed erosions in the hands of five of the six patients with normal radiographs.

Leidersnaider et al.^21^ examined joint involvement in 30 patients (26 women) with CCA with ages ranging from 32 to 73 years (mean age 54.7 ± 10.0 years) using physical examination, ultrasonography, and MRI. The mean time from diagnosis to ultrasonography was 336.1 ± 251.7 days, ranging from 53 to 911 days, and the mean time from diagnosis to MRI was 337.5 ± 251.7 days, ranging from 54 to 913 days. The most common sites of involvement identified by ultrasonography were the metacarpophalangeal (MCP) joints (83%), extensor and flexor sheaths (60% each), and radiocarpal/distal radioulnar joint/proximal carpal joints (53%). MRI revealed fluid distension of the extensor sheaths (63%), synovitis of the MCP joints, radiocarpal/ distal radioulnar joint/proximal carpal recesses (57%), and fluid distension of flexor sheaths (40%). Both imaging techniques also detected findings such as increased vascular flow, median nerve changes, subcutaneous edema, bone marrow edema, and erosions.

Mogami et al.^22^ performed a cross-sectional observational study of 50 sequential cases of CCA in patients who underwent ultrasonography and radiography of the hands and wrists. Of these 50 patients, 44 (88%) were female. The mean age was 56.9 ± 13 years. The mean interval between disease onset and the ultrasound and radiographic examinations was 3.97 ± 1.09 months. The study compared the rheumatological manifestations with the radiological and ultrasonographic findings in patients with joint pain and stiffness. Among the sonographic alterations observed in the hands and wrists, small joint synovitis was present in 42 patients (84%), and effusion and/or synovial thickening in the radiocarpal/radioulnar/proximal intercarpal regions were present in 37 patients (74%). Hand and wrist radiographs revealed marginal erosion in only 1 (2%) patient, but 19 (38%) had radiographs that showed abnormalities, including osteopenia in 9 patients (18%), osteoarthritis in 7 patients (14%), and soft tissue swelling in 5 patients (10%)^22^.

## DISCUSSION

CHIKF is an emerging arboviral disease which has an important public health impact due to its debilitating musculoskeletal symptoms. Joint damage is a prominent feature of CHIKV infection and can result in long-term morbidity and disability^4^. CHIKV targets the synovial tissue, resulting in a spectrum of musculoskeletal manifestations. The pathogenesis of joint damage in CHIK infection is multifactorial and involves a complex interplay between viral factors and host immune responses^5^. Viral replication within joint tissues triggers an inflammatory cascade, leading to the release of pro-inflammatory cytokines, chemokines, and matrix metalloproteinases. These mediators contribute to synovial inflammation, cartilage degradation, and bone erosion^6^ ^-^ ^8^. The adaptive immune response plays a crucial role in joint pathology, with both cellular and humoral immune mechanisms implicated. T cells, B cells, macrophages, and dendritic cells within the synovium, perpetuating joint inflammation and tissue damage. Other autoimmune and host genetic factors may also influence the severity and persistence of joint symptoms^9^.

The pathogenesis of CCA has similarities to the pathogenesis of RA and the studies of CCA-associated joint damage reviewed demonstrated clinical similarities between CCA and RA. This is important because the concept of treat-to-target to achieve clinical remission or low disease activity is well established in RA management. The goal of this strategy in RA is to prevent joint damage and disability through early and aggressive intervention^8^ ^,^ ^23^. Disease modification to prevent structural damage to cartilage and bone has become a key focus of modern treatment. In the past 30 years, DMARDs, rather than symptomatic agents, have become the standard of care^7^. In RA, increasing evidence supports the use of strict disease control measures to enhance outcomes^7^ ^,^ ^8^ ^,^ ^23^.

The available evidence suggests that CCA results in progressive, irreversible joint destruction, comparable to RA; however, current studies are limited by the small sample sizes and the low level of evidence^4^ ^,^ ^6^ ^,^ ^9^ ^,^ ^24^. In our clinical practice, we have observed that many patients with CCA develop joint deformities similar to those observed in patients with RA. However, the current recommendations for CCA treatment resemble RA treatment recommendations from three decades ago. Despite the limited number of studies on joint damage caused by CHIKV, the available evidence demonstrates that CHIKV can cause joint damage similar to that of RA. CHIKV causes epidemics worldwide, resulting in chronic joint pain and deformities. However, current treatment recommendations do not take this risk into account. Better characterization of the spectrum of CCA through well-designed, larger longitudinal cohort studies is needed to evaluate joint damage and the potential benefits of DMARDs in CCA.

## CONCLUSION

Published studies provide evidence that CHIKV infection causes CCA with significant joint damage similar to that of RA; however, clinical trials are needed to assess the optimal treatment of CCA. The pathogenesis of CCA involves complex interactions between viral factors and host immune responses, resulting in synovial inflammation, cartilage degradation, and bone erosion, with similarities to the pathogenesis of RA. However, the current treatment recommendations for these diseases differ. Current treatment approaches for CCA are reminiscent of the RA recommendations from several decades ago, with a focus on symptomatic relief rather than disease modification. This systematic review underscores the limited but emerging research on joint damage caused by CHIKV infection and the potential benefit of early intervention using DMARDs. The paradigm shift in RA treatment toward early and aggressive therapy to halt disease progression raises the question of whether a similar approach is warranted for CCA. With the increasing prevalence of CHIKF worldwide and its long-term impact on joint health, further investigations and larger longitudinal studies are crucial to establish an optimal management strategy, and to assess whether early use of DMARDs is effective at mitigating joint damage and improving patient outcomes.
