# Supplementary material for: Bone erosions and joint damage caused by chikungunya virus: a systematic review
Source: Rev Soc Bras Med Trop. 2024 Apr 5;57:e00404-2024. doi: 10.1590/0037-8682-0433-2023 (PMC11000508; doi:10.1590/0037-8682-0433-2023)
Supplement: Supplementary file 1 [file 1678-9849-rsbmt-57-e00404-2024-supp1.pdf]

S1_Table. Search strategies

Search Date	Data base	Strategy used	Number of Studies
June 06, 2023	MEDLINE (PubMed)	("Chikungunya Fever"[Mesh] OR "Chikungunya Virus Infection" OR "Chikungunya Fever" OR "Chikungunya virus"[Mesh] OR "Chikungunya" OR "CHIK" OR "CHIKV") AND ("Bone erosion" OR " Joint damage")	Chikungunya fever [Mesh]+ bone erosion = 0<br>Chikungunya fever [Mesh]+ joint damage = 1<br><br>Chikungunya fever + joint damage = 1<br>Chikungunya fever + bone erosion= 0<br><br>Chikungunya Virus Infection + bone erosion = 1<br>Chikungunya Virus Infection + joint damage = 1<br><br>Chikungunya virus [Mesh]+ bone erosion = 0<br>Chikungunya fever [Mesh]+ joint damage = 1<br><br>Chikungunya + joint damage = 1<br>Chikungunya + bone erosion = 0<br><br>Chik + joint damage = 0<br>Chik + bone erosion = 0<br><br>Chikv+ joint damage = 1<br>Chikv + bone erosion = 0
June 06, 2023	The Cochrane Library	# 1- MeSH descriptor: [Chikungunya Fever] explodes all trees<br># 2- MeSH descriptor: [Chikungunya virus] explodes all trees<br>#3- #1 AND #2	1- 52<br>2- 18<br>3- 70
June 06, 2023	SCOPUS	((TITLE-ABS-KEY ("Chikungunya Fever") OR TITLE-ABS-KEY ("Chikungunya virus"))) AND ((TITLE-ABS-KEY (" Bone erosion ") OR TITLE-ABS-KEY ("Joint damage")))	Chikungunya virus + bone erosion = 8<br>Chikungunya fever + bone erosion = 5<br>Chikungunya fever + Joint damage = 5<br>Chikungunya virus + Joint damage = 5

June 06, 2023	EMBASE	('chikungunya'/exp OR 'chikungunya') AND ('bone erosion'/exp OR 'bone erosion' OR (('bone'/exp OR bone) AND ('erosion'/exp OR erosion))) OR ('chikungunya'/exp OR 'chikungunya') AND ('joint damage' OR (('joint'/exp OR joint) AND damage))	11 + 7
