# Supplementary material for: Bone erosions and joint damage caused by chikungunya virus: a systematic review
Source: Rev Soc Bras Med Trop. 2024 Apr 5;57:e00404-2024. doi: 10.1590/0037-8682-0433-2023 (PMC11000508; doi:10.1590/0037-8682-0433-2023)
Supplement: Supplementary file 2 [file 1678-9849-rsbmt-57-e00404-2024-supp2.pdf]

**S2_Table. Data extraction**

| 1st author (year) [ref] | Country and year of study | Study Design | N Total / Gender | Age | Diagnosis type/ Date extraction type | Outcomes | Comments |
|---|---|---|---|---|---|---|---|
| Eyer-Silva et al (2016) | Brazil, 2016 | Case report | n=1<br>1 M | 36 | 1 confirmed Chikungunya<br><br>Clinical and laboratory Examinations (RT-PCR) | severe acute arthritis in a finger joint previously damaged by trauma | |
| Guillot et al (2020) | France (2018 to 2019) | longitudinal observational monocentric study | N=30/<br>20 F<br>10 M | F (66 [49–83])<br>M (33 [17–51]) | 30 confirmed Chikungunya<br><br>Clinical and laboratory Examinations | 7 (23.3%) Radiographic structural damage (joint erosions and/or joint space narrowing) | After the 2005–2006 outbreak, 159 patients were first referred to a rheumatologist for post-chikungunya chronic musculoskeletal pain, 73 of them were diagnosed with classifiable chikungunya-related chronic inflammatory rheumatic diseases. Thirty of these 73 patients were clinically evaluated by a second rheumatologist in 2018–2019. |
| Manimunda et al (2010) | India (June 2008 to April 2009) | longitudinal follow-up study | N=203<br><br>107 F<br>96 M | F (median 36.5; IQR 28–48)<br>M (median 33.5; IQR 22.5–50). | 203 confirmed Chikungunya<br><br>Clinical and laboratory Examinations (Anti-CHIKV IgM antibody) | In 20 patients the presence of RF and anti-CCP was tested.<br><br>(5/20) patients showed radiolucent lesions on the X-ray suggestive of bony erosion.<br>In MRI:<br>(16/20) patients: joint effusion<br>(4/20) patients: erosion;<br>(7/20) patients: marrow oedema/erosion;<br>(4/20) patients: synovial thickening<br>(2/20) patients: tenosynovitis<br>(3/20) patients: tendinitis. | A subpopulation of the patients with joint pain (20/94) was tested for rheumatoid factor (RF) and anti-cyclic citrullinated peptide (anti-CCP) antibody, and the joints were imaged by X-ray and magnetic resonance imaging (MRI). Findings suggestive of degenerative disease of the joints, tearing of menisci and cruciate ligaments were also observed. |

| | | | | | | | |
|---|---|---|---|---|---|---|---|
| Bouquillard and Combe (2009) | France (February 2006 to July 2007) | a prospective follow-up study | N = 21<br><br>13 F<br>8 M | Mean age 57.3 ± 12.2 years) | 21 confirmed Chikungunya<br><br>Clinical and laboratory Examinations (IgM or IgG antibodies) | Radiographic analysis of hands and feet<br>At diagnosis:<br><br>5 (23.8%): erosions<br>12 (57.1%): joint space narrowing<br><br>At ~24 months' follow-up<br><br>17 (81%): erosions<br>17 (81%): Joint space narrowing | Patients with possible RA after CHIKV infection and RA according to the 1987 American College of Rheumatology Criteria.<br>The mean delay between CHIK fever onset and RA diagnosis was 10 months (range 4–18). |
| Leidersnaider et al (2021) | Brazil (January 2018 to March 2020. | cross-sectional study | N=30<br>26 F<br>4 M | a mean ± SD 54.7 ± 10.0 years. | 30 confirmed Chikungunya<br><br>IgG serology in 23 patients (76.7%), IgM and IgG serology in 3 (10%), IgM serology alone in 2 (6.6%) and PCR in 2 (6.6%) | MRI:<br>11 (36.7%): bone marrow edema<br>8 (26.2%): bone erosion<br>5 (16.7%): subcutaneous edema<br><br>US:<br>25 (83.3%): effusion or synovial thickening<br>1 (3.3%): bone erosion | Mean elapsed time between the diagnosis and US examination was 336.1 ± 251.7 days,<br>For MRI, the mean elapsed time was 337.5 ± 251.7 days |
| Mogami et al (2017) | Brazil (June to October 2016) | cross-sectional observational study | N=50<br>44 F<br>6 M | mean age was 56.9 6 13 years | laboratory diagnosis of<br>CHIKF (IgM/IgG) | US:<br>42 (84%): joint synovitis<br>37 (74%): effusion and/or synovial thickening<br><br>Radiographic Findings:<br>31 (62%): Normal<br>9 (18%): Periarticular or diffuse osteopenia<br>7 (14%): Osteoarthritis<br>5 (10%): Increase of soft parts<br>4 (8%): Nonspecific findings<br>1 (2%): Marginal erosions | ultrasound exams were performed<br>3.97 6 1.09 months after the onset of the disease |
| Malvy et al (2009) | France (2007) | Case report | N=1<br>M | 60 years | IgM-capture and an IgG-capture enzyme-linked | <br>Radiography:<br>Subchondral defect of the 2nd and 3rd right proximal interphalangeal | Transcriptomic and protein analysis revealed individual genes which could be implicated in the |

| | | | | | | | |
|---|---|---|---|---|---|---|---|
| | | | | | immunoabsorbent assay | finger joints as well as of the 3rd, 4th and 5th left distal interphalangeal joints.<br><br>MRI:<br>Bilateral periostal inflammation<br>Oedematous carpitis<br>Carpis synovitis<br>Bone destruction<br>Intra-articular swelling | pathogenesis of chronic arthritis |
| Mizuno et al (2011) | Japan<br>(2006 to 2009) | Case report | N=6<br>4 F<br>2 M | F 36, 39,39,56<br>M 30,52 | IgM-capture enzyme-linked immunoassay and a plaque reduction neutralizing antibody assay | MRI:<br>1 patient with extensive intermediate-signal-causing carpal styloid erosions | None |
| | | | | | | - | |
| | | | | | | | |
